# Development of Molecular Mechanisms and Their Application on Oncolytic Newcastle Disease Virus in Cancer Therapy

**DOI:** 10.3389/fmolb.2022.889403

**Published:** 2022-07-04

**Authors:** Fang Huang, Chuanjing Dai, Youni Zhang, Yuqi Zhao, Yigang Wang, Guoqing Ru

**Affiliations:** ^1^ Cancer Center, Department of Pathology, Zhejiang Provincial People’s Hospital (Affiliated People’s Hospital, Hangzhou Medical College), Hangzhou, China; ^2^ College of Life Sciences and Medicine, Xinyuan Institute of Medicine and Biotechnology, Zhejiang Sci-Tech University, Hangzhou, China; ^3^ Department of Laboratory Medicine, Tiantai People’s Hospital, Taizhou, China

**Keywords:** Newcastle disease virus, oncolytic virotherapy, tumor, apoptosis, antitumor immunity

## Abstract

Cancer is caused by the destruction or mutation of cellular genetic materials induced by environmental or genetic factors. It is defined by uncontrolled cell proliferation and abnormality of the apoptotic pathways. The majority of human malignancies are characterized by distant metastasis and dissemination. Currently, the most common means of cancer treatment include surgery, radiotherapy, and chemotherapy, which usually damage healthy cells and cause toxicity in patients. Targeted therapy is an effective tumor treatment method with few side effects. At present, some targeted therapeutic drugs have achieved encouraging results in clinical studies, but finding an effective solution to improve the targeting and delivery efficiency of these drugs remains a challenge. In recent years, oncolytic viruses (OVs) have been used to direct the tumor-targeted therapy or immunotherapy. Newcastle disease virus (NDV) is a solid oncolytic agent capable of directly killing tumor cells and increasing tumor antigen exposure. Simultaneously, NDV can trigger the proliferation of tumor-specific immune cells and thus improve the therapeutic efficacy of NDV in cancer. Based on NDV’s inherent oncolytic activity and the stimulation of antitumor immune responses, the combination of NDV and other tumor therapy approaches can improve the antitumor efficacy while reducing drug toxicity, indicating a broad application potential. We discussed the biological properties of NDV, the antitumor molecular mechanisms of oncolytic NDV, and its application in the field of tumor therapy in this review. Furthermore, we presented new insights into the challenges that NDV will confront and suggestions for increasing NDV’s therapeutic efficacy in cancer.

## Introduction

Cancer seriously threatens human health due to its high incidence and mortality and is the second cause of death globally, exceeded only by cardiovascular diseases (Moliner et al., 2019; Xia et al., 2022). In 2020, there were an estimated 19.3 million new cancer cases worldwide and nearly 10 million cancer-related deaths (Sung et al., 2021). Cancer’s high mortality rate is mainly because patients with early cancer have no apparent symptoms and are already in the late stage or metastatic stage when diagnosed (Huang et al., 2017; Regel et al., 2020). Tumor cells evade immune system surveillance and inhibit the immune response due to high mutagenicity (Park et al., 2020). At the same time, uncontrolled cancer cells invade the tissue, eventually leading to organ failure and even death (Fares et al., 2020). Currently, surgery, radiotherapy, and chemotherapy are the main methods for cancer treatment (Moo et al., 2018; Yahya and Alqadhi, 2021). Although surgery, radio-/chemotherapy, and targeted therapy can help some patients with early tumors, most therapy methods for individuals are terminated because of severe side effects (Boshuizen and Peeper, 2020). Cancer prognosis is still not optimistic; therefore, an essential question in cancer therapy as to how to improve cancer patients’ survival rate effectively remains unexplored.

In recent years, several approaches have been developed for cancer therapy, such as immune checkpoint–based therapy (Chen et al., 2021), targeting circular RNAs (Chen et al., 2019), chimeric antigen receptor T (CAR-T) cell therapy (Adachi et al., 2018), and CRISPR/Cas9-based therapy (Zhen and Li, 2019). But all of these approaches have some limitations, which include off-target effects for targeted therapy, inefficiency of monotherapy, and unpredictable or predictable side effects (Zugazagoitia et al., 2016). An oncolytic virus (OV) is a promising cancer treatment strategy. OV is a useful therapeutic reagent that identifies and destroys malignant cells after a recurring viral infection (Martin and Bell, 2018; Raja et al., 2018; Leber et al., 2020). The lytic products after tumor dissolution can reverse the tumor microenvironment, promote the recruitment of immune cells, and further activate the antitumor immune response (Mahasa et al., 2017; Harrington et al., 2019). Several viruses, including the Newcastle disease virus (NDV), vaccinia virus, adenovirus, reovirus, herpes simplex virus, and measles virus, are being widely studied to treat various types of advanced cancer (Mondal et al., 2020; Goradel et al., 2021). Talimogene laherparepvec (T-VEC), a genetically engineered herpes simplex virus, is the first OV approved to treat advanced melanoma by the US FDA (Liu et al., 2003; Andtbacka et al., 2019). However, due to the heterogeneity of cancer tissue and the complexity of cancer cells, a single type of OV is not enough to destroy all cancer cells (Lawler et al., 2017; Martin and Bell, 2018). Some cancer cells and non-transformed supporting cells may be resistant to certain OVs (Alvarez-Breckenridge et al., 2013; Pol et al., 2016). Based on these, a single type of viral therapy may not be effective against all types of cancer (Kwan et al., 2021). Therefore, we believe that the combination of OV therapy and other cancer therapies will be significant for cancer patients (Dai et al., 2022).

NDV is a natural avian–derived virus (Sinkovics and Horvath, 2000), and its infection is a highly contagious disease that causes enormous economic losses to the poultry industry worldwide (Ganar et al., 2014; Susta et al., 2018). NDV has been developed as an oncolytic agent or a vaccination vector over the last 20 years due to its intrinsic oncolytic ability (Molouki and Peeters, 2017; Hu et al., 2020; Vannini et al., 2021). Compared with other OVs, oncolytic NDV has inherent antitumor advantages (Meng et al., 2021). Natural NDV strains exhibit an antitumor effect in human cancer cells and cause oncolysis without harming the normal cells (Yurchenko et al., 2019; Burman et al., 2020). In addition to causing direct damage to host cells through viral infection and replication, NDV activates multiple signaling pathways, triggering autophagy, inflammation, and apoptosis (Cuadrado-Castano et al., 2015; Li et al., 2019; Gong et al., 2021). It also activates antitumor immune responses, thus assisting viral replication (Wang et al., 2020; Zhan et al., 2020; Kan et al., 2021). With the constant maturity of the reverse genetic operating system of NDV (Peeters et al., 1999; Römer-Oberdörfer et al., 1999; Nettelbeck et al., 2021), an increasing number of transgenic NDVs are identified, which makes the application of NDV a new stage in cancer therapy. A genetically engineered NDV strain (NDV-F3aa) is effective in the experimental treatment of a gastric tumor peritoneal model without significant toxicity, and in some cases, it may completely cure gastric tumors (Song et al., 2010). Combining NDV therapy with other cancer therapies also provides new ideas for cancer treatment (Schirrmacher and Fournier, 2014; Xu et al., 2021). Hence, the NDV represents broad prospects for cancer treatment.

This review will concentrate on the biology, process of infection, and replication of NDV in cancer cells and the primary molecular mechanism of NDV oncolysis, and its preclinical and clinical applications in diverse cancers. In addition, we will highlight the limitations of NDV in clinical research and share our new insights into the use of NDV in cancer therapy.

## Newcastle Disease Virus Biology

**FIGURE 1:**
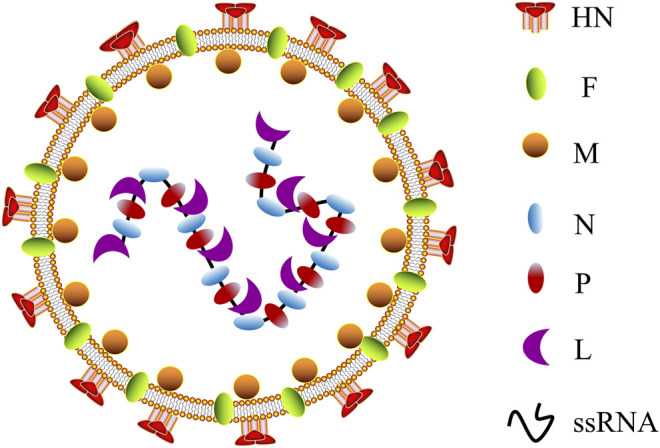
Schematic representation of NDV morphology. NDV, Newcastle disease virus; HN, hemagglutinin–neuraminidase; F, fusion protein; M, matrix protein; N, nucleocapsid protein; P, phosphoprotein; L, RNA-dependent large polymerase protein; ssRNA, single-stranded RNA.

Twelve different serotypes of avian paramyxoviruses (APMVs) have been reported up to date (Gogoi et al., 2017). NDV is the most characterized member of the genus *Avulavirus* in the family of Paramyxoviridae (APMV-1) (Kapczynski et al., 2013). It is an RNA virus with diameters ranging from 100 to 500 nm, enclosed by a viral lipid membrane (Kapczynski et al., 2013; Nagai et al., 1989). NDV was first identified as a valuable virus for virulence studies in the 1970s (Gogoi et al., 2017; Cassel and Garrett, 1965). According to their pathogenicity and virulence in infected chickens, NDV strains are classified as lentogenic (avirulent), mesogenic, and velogenic (fully virulent) (Sinkovics and Horvath, 2000; Dimitrov et al., 2016). NDV contains a negative single-stranded RNA (ssRNA) genome of approximately 15.2 kb that consists of a leader (55 nucleotides) and trailer (114 nucleotides) terminal sequences (Nagai et al., 1989; Bello et al., 2020), which encode six different structural proteins: hemagglutinin–neuraminidase (HN), nucleocapsid (N) protein, fusion (F) protein, phosphoprotein (P) protein, matrix (M) protein, and RNA-dependent large polymerase (L) protein (Figure 1). V and W proteins are auxiliary and exist only in virus-infected cells. The V protein is an IFN antagonist and plays a vital role in the virulence of NDV (Alamares et al., 2010). Notably, in the NDV genome, each gene encodes a single protein and is characterized by a coding sequence flanked by highly conserved gene start (GS) and gene end (GE) transcriptional signals (Munir et al., 2012).

The viral N protein, P protein, and L protein bind to the viral RNA genome to form a ribonucleoprotein complex (RNP), essential for virus replication (Yusoff and Tan, 2001). The M protein is located in the layer below the virus lipid membrane and participates in virus assembly and budding (Nagai et al., 1989). HN and F proteins are located on the virus membrane’s outer surface, where they join with the host cell’s lipid bilayer membrane to form a viral shell. In addition, HN and F proteins jointly mediate viral attachment and fusion on the cell surface (Fournier et al., 2004). The fusion of virus and host cell must be completed through the F protein and HN protein participation, and the cleavage site of virus F protein (Fcs) is the critical factor (Peeters et al., 1999; Seal et al., 2000). Simultaneously, antibodies F and HN are the significant components that resulted in vaccine-inducing body protection, following vaccination of avian or non-avian species (Xiao et al., 2012; Dey et al., 2014), revealing the potential of NDV as a vaccine vector resistant to the animal and human disease. Currently, the LaSota and Hicher B1 vaccine strains have been widely used as a live NDV vaccine throughout the world (Carrasco et al., 2016; Dey et al., 2017). The strains are naturally occurring lentogenic strains that are highly expressed in embryonated chicken eggs and elicit a significant immune response (Ginting et al., 2017). One of the advantages of NDV as an oncolytic agent is that both lytic and non-lytic strains of NDV can fast-replicate in all species of avian and multiple human cancer cells (Lam et al., 2011; Zamarin and Palese, 2012), resulting in effective cell lysis and offering substantial protection from disease.

## Newcastle Disease Virus Dissolves Tumor and Activates an Antitumor Immune Response

**FIGURE 2:**
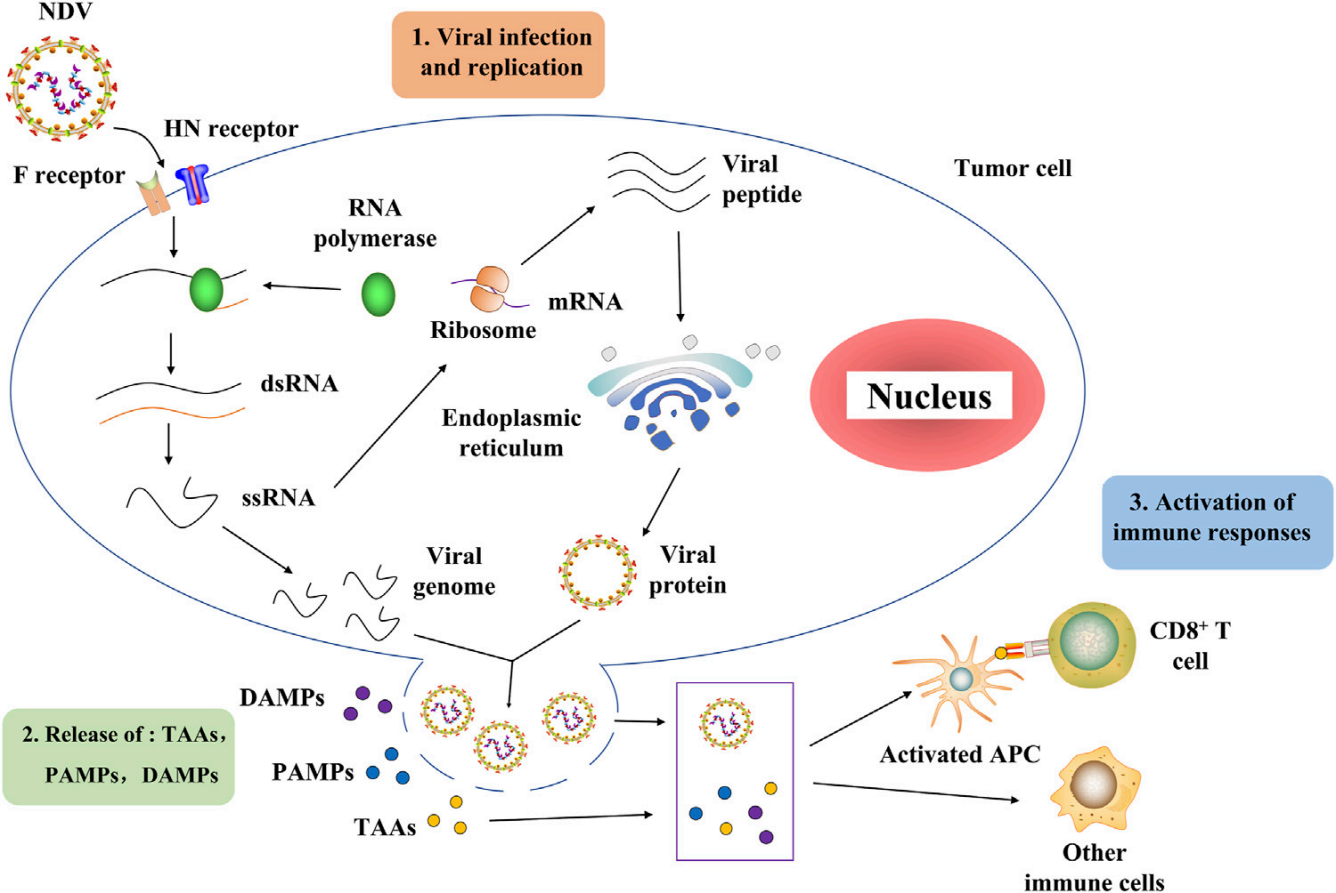
Process of NDV through which it infects tumor cells and activates the host immune system. NDV exerts its antitumor effect mainly in two stages. In the first stage, the NDV binds to the sialic acid receptor on the surface of tumor cells through the HN protein, and then protein F initiates the fusion of the viral and host cell membranes. Then, the viral RNA polymerase transcribes the viral negative single-stranded RNA into positive single-stranded RNA as a template for mRNA and protein synthesis. The rough endoplasmic reticulum processes surface proteins F and HN, assembled on the host cell membrane and germinated to produce new virions that begin a new round of tumor cell infection. In the second stage, the virus-mediated direct oncolysis leads to the release of TAAs, PAMPs, and DAMPs that activate APCs, including dendritic cells capable of antigen cross-presentation. Activated APCs activate immune cells, resulting in the generation of CD4^+^ T cells, CD8^+^ T cells, and NK cells directed toward tumor and viral antigens. NDV, Newcastle disease virus; HN, hemagglutinin–neuraminidase; F, fusion protein; dsRNA, double-stranded RNA; ssRNA, single-stranded RNA; TAAs, tumor-associated antigens, PAMPs, pathogen-associated molecular patterns, DAMPs, danger-associated molecular patterns; APCs, antigen-presenting cells.

The NDV oncolytic properties originate from its capacity to proliferate in cancer cells (Shobana et al., 2013). Further research showed that it might be related to the deficiency of the interferon (IFN) system in tumors (Stojdl et al., 2000). H. Song et al. discovered that NDV enters the cell through a pH-independent direct fusion of its envelope to the host membrane *via* receptor-mediated endocytosis (Sánchez-Felipe et al., 2014). The process of NDV infection and replication in tumor cells is described as follows (Moliner et al., 2019). NDV binds to the sialic acid receptor on the surface of tumor cells through the HN protein, and then, protein F initiates the fusion of the viral and host cell membranes (Song et al., 2019; Xia et al., 2022). Viral RNA polymerase transcribes the viral negative single-stranded RNA into positive single-stranded RNA as a template for mRNA and protein synthesis (Burman et al., 2020; Sung et al., 2021). The rough endoplasmic reticulum processes surface proteins F and HN, assembled on the host cell membrane and mature to produce new virions that start a new round of tumor cell infection (Cuadrado-Castano et al., 2015). Importantly, virus-mediated direct oncolysis causes the release of tumor-associated antigens (TAAs), pathogen-associated molecular patterns (PAMPs), and danger-associated molecular patterns (DAMPs). These can activate antigen-presenting cells (APCs), including antigen-cross-presenting dendritic cells (DCs). Activated APCs then activate the immune cells, resulting in the generation of CD4^+^ T cells, CD8^+^ T cells, and NK cells directed toward tumor and viral antigens (Burman et al., 2020; Schirrmacher and Fournier, 2014) (Figure 2). It is worth mentioning that NDV does not replicate in the normal cells of non-avian hosts (Fiola et al., 2006).

NDV is an effective oncolytic agent. The oncolytic properties of NDVs are also correlated with the pathogenic classification of NDV strains (lytic or non-lytic) (Dey et al., 2014). It has been found that mesogenic and velogenic NDVs are lytic while lentogenic NDV is non-lytic (Fournier et al., 2012; Ganar et al., 2014). Velogenic NDV kills cancer cells rapidly because they destroy the cytoplasmic membrane of infected cells (Wu et al., 2014). Lytic NDV exhibits multi-loop replication, whereas the non-lytic virus exhibits only single-loop replication (Fournier et al., 2012). In addition, the replication process of NDV takes place in the cytoplasm. This replication mode prevents the virus from integrating with the host genome or recombining with the human virus itself (Ganar et al., 2014). Therefore, NDV is non-pathogenic to humans and thus relatively safe with no side effects, which is a significant advantage of NDV as an oncolytic agent.

## Multiple Antitumor Molecular Mechanisms of Newcastle Disease Virus

The induction of apoptosis, autophagy, necroptosis, and immunogenic death (ICD), as well as the stimulation of the immune system, are among NDV’s oncolytic processes (Cuadrado-Castano et al., 2015; Zhang et al., 2018; Shao et al., 2019; Kan et al., 2021). The antitumor mechanism of NDV is briefly described in the following section.

### Newcastle Disease Virus Activates the Immune Response

As mentioned earlier, NDV selectively infects tumor cells and rapidly replicates in tumor cells to directly dissolve tumors (Fiola et al., 2006). Significantly, NDV oncolysis reshapes the tumor microenvironment (TME), transforming cold tumors into hot tumors (Burman et al., 2020). This process is beneficial for immune cells to infiltrate tumors. On the one hand, NDV induces the release of the risk-related molecular model of strong antitumor immunity after oncolysis, such as TAAs, PAMPs, and DAMPs (Figure 2). These key risk–related molecular models can activate not only some innate immune cells (NK cells) but also tumor-specific T cells (CD4^+^ and CD8^+^ T cells) and recruit APCs into the tumor to initiate an immune response (Schild et al., 1989; Ricca et al., 2018). Remarkably, upregulation of many immune checkpoint molecules (CTLA-4 and PD-1) has been observed on CD4^+^ and CD8^+^ T cells in recent years (Zamarin et al., 2014; Nakao et al., 2020). This suggests the possibility of combining NDV and immune checkpoint inhibitors to break immune resistance. On the other hand, the activated non-specific immune cells kill and devour infected tumor cells that are not lysed or resistant to viral oncolysis (Fuertes et al., 2011); when the inflammatory response to NDV infection helps the immune system clear tumors, it also causes immune cells to clear NDV, limiting antitumor effects (Buijs et al., 2014). As a result, developing NDV-based cancer regimens necessitates striking a balance between appropriate viral replication, tumor lysis, and immune response activation.

### Newcastle Disease Virus Mediates the Apoptosis Pathway

**FIGURE 3:**
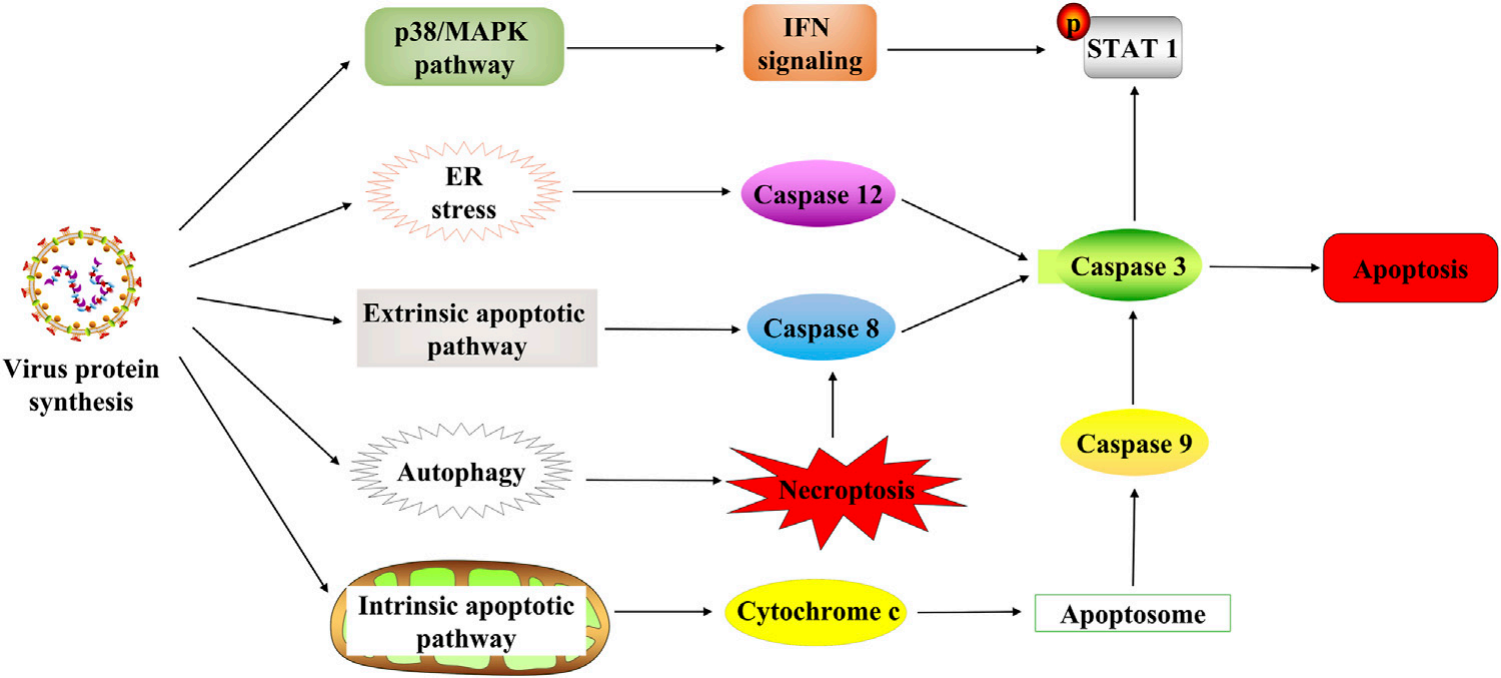
NDV-induced cell death in tumor cells. NDV regulates cell death through multiple mechanisms after infection with tumor cells, including the p38/MAPK pathway, ER stress, apoptosis pathway, and autophagy pathway. STAT1, signal transducer and activator of transcription 1; ER, endoplasmic reticulum.

Apoptosis usually occurs as a defense mechanism, such as in the immune response or when cells are damaged by harmful substances (Norbury and Hickson, 2001); while NDV can induce apoptosis to dissolve tumors (Figure 3). The oncolytic selectivity of NDV on tumor cells depends on tumor cell resistance to apoptosis (Mansour et al., 2011). NDV infection induces the apoptosis of tumor cells mainly through the exogenous and the endogenous pathways (mitochondrial-related pathways) (Liao et al., 2017; Song et al., 2019). Tumor cells infected with NDV can cause the release of cytokines such as IFN-α, IFN-β, and TNF-α, which activates the NF-kB signaling pathway, which in turn stimulates the exogenous apoptotic pathway (Wilden et al., 2009; Elankumaran et al., 2010). Furthermore, compared with normal cells that can secrete both IFN-α and IFN-β, tumor cells infected with NDV strain AF2240 only release IFN-β (Ch’ng et al., 2013). A study by Ghrici et al. (2013)revealed that the mitochondrial-related pathway may be the central activator in NDV-induced apoptosis. They found that AF2240 infected cells activated the opening of mitochondrial transition pores, resulting in the activation of caspase-8 and then the viral NP gene expression. Therefore, the apoptosis-inducing effect of NDV may be independent of virus replication and protein synthesis. In 2015, the p38/MAPK pathway was fully elucidated in NDV-mediated apoptosis (Ch’ng et al., 2015). In NDV-infected tumor cells, phosphorylation of p38 mitogen–activated protein kinase (MAPK) was increased by proinflammatory cytokines during infection. This cytoplasmic stimulation degrades the inhibitor of NF-κB, thus releasing NF-κB (Lawrence, 2009). Furthermore, NDV stimulates the immune system to produce cytokines such as IFN-λ (Bu et al., 2016a), which targets phosphor-STAT1 degradation to block IFN-I signaling (Qiu et al., 2016) and exerts an antitumor effect. Thus, the IFN responsiveness may provide a detection indicator for virotherapy (Pease and Kratzke, 2017). ER stress contributes to the antiviral response to NDV by inducing and increasing apoptosis (Bu et al., 2016b; Shokeen et al., 2021). ER stress reduces viral replication due to eIF2α phosphorylation and induces an alternative caspase 12-dependent programmed cell death response (Bu et al., 2016b; Yan et al., 2018).

### Newcastle Disease Virus Regulates Autophagy

Autophagy is an evolutionarily conserved intracellular process that influences cellular immune responses (Su et al., 2015). At the same time, autophagy is associated with various diseases such as cancer (Smith and Macleod, 2019). The NDV infection also induces tumor-specific autophagy (Figure 3). Many recent studies have focused on the critical role of autophagy in the viral treatment of cancers (Huang et al., 2018; Mattoscio et al., 2018). In addition, NDV exploits the autophagic processes to facilitate their replication, enhancing oncolysis against tumor cells, often leading to tumor necroptosis (Cheng et al., 2016). Furthermore, NDV promotes viral replication *via* autophagy by inhibiting caspase-dependent apoptosis in cancer cells. Because NDV-induced apoptosis, host immune response, and autophagy affect NDV replication in cancers, it is reasonable to conclude that apoptosis and autophagy are mutually regulated (Pei et al., 2016; Ravegnini et al., 2017; Zhang et al., 2017). Previous research study has revealed that the oncolytic NDV strain NDV/FMW promotes apoptosis in lung cancer cells and facilitates oncolysis in resistant tumor cells suggesting a link between apoptosis and autophagy. Its effect is amplified by the pharmacological regulation of autophagy (Hu et al., 2015). Thus, NDV induces autophagy in apoptotic pathways through the regulation of autophagic activity (Figure 3). Then autophagy inhibits apoptosis and contributes to NDV infection in cancer cells, activating immunity responses *in vivo* and eventually killing the tumor cells.

## Preclinical Application of Newcastle Disease Virus in Various Cancers

NDV has been widely used in preclinical research as a novel anticancer drug for numerous solid tumors and resistant cancers (Table 1). Combining NDV therapy with various cancer medications may fully activate the innate and adaptive antitumor immunity based on the inherent oncolytic capabilities of NDV and its interaction with the immune system. This part summarizes NDV’s use in the preclinical treatment of various cancers.

**TABLE 1:** NDV strains in the treatment of different cancers in preclinical trials.

Cancer type	NDV strain	Combination	Outcome	Reference
Gastric cancer	NDV (F3aa)	—	There was no gross tumor in six (40%) NDV-treated mice, and the nodules were significantly smaller than untreated mice	Song et al. (2010)
	rL-hIFN-λ1	—	rL-hIFN-λ1 inhibited the growth of gastric cancer cell lines which contained the IFNλ-R1 receptors and accelerated cancer cell apoptosis	Bu et al. (2016a)
	NDV-D90	—	NDV-D90 induced gastric cancer cell apoptosis and reduced cell invasion in a dose-dependent manner in the highly differentiated gastric cancer cell line	Sui et al. (2017)
Liver cancer	rNDV-18HL	—	rNDV-18HL selectively replicated in orthotopic HCC xenografts, which induced tumor necrosis, reduced intrahepatic metastasis, and prolonged the survival in mice	Wei et al. (2015)
	NDV/Anh-IL-2	—	NDV/Anh-IL-2-treated animals exhibited significantly increased numbers of tumor-infiltrating lymphocytes	Wu et al. (2016)
	LaSota	Fludarabine	The combination of fludarabine with NDV significantly improved NDV-mediated antitumor immunity and prolonged survival in a mouse model of HCC	Meng et al. (2019)
	AF2240 and V4-UPM	5-Fluorouracil	The combination of NDV and 5-fluorouracil had greater antitumor efficacy than NDV or 5-FU alone	Assayaghi et al. (2019)
Lung cancer	rL-RVG	—	The growth of A549 cells in the rL-RVG group was inhibited more effectively than those infected with the wild-type NDV strain	Yan et al. (2015)
	NDV/FMW	Chloroquine	Treatment of spheroids with the autophagy inhibitor chloroquine increased NDV/FMW-induced cytotoxicity	Hu et al. (2015)
Breast cancer	AMHA1	—	NDV is replicated efficiently in cancer cells and spares normal cells and induces morphological changes and apoptosis in breast cancer cells	Al-Ziaydi et al. (2020a)
	AF2240	—	Breast cancer cells in allotransplanted mice treated with AF2240 showed a noticeable inhibition of tumor growth and induced apoptotic-related cytokines	Raihan et al. (2019)
	AMHA1	2-Deoxyglucose	The combination therapy group induced the highest rate of tumor growth inhibition (100%), followed by the NDV group (96.8%)	Zhao et al. (2008)
Cervical cancer	LaSota	—	NDV treatment significantly reduced the viability of cervical cancer cells and inhibited tumor growth by inducing ROS-mediated apoptosis	Keshavarz et al. (2020a)
	NDV HB1	—	Peritumoral injection of NDV oncolysate induces robust antitumor immune responses in the mouse model	Mozaffari Nejad et al. (2021)
Colorectal cancer	R2B Mukteshwar	—	Significant tumor lytic activity was evident when R2B Mukteshwar was injected *via* the intratumoral route	Sharma et al. (2017)
	rAF-IL12	—	rAF-IL12 regulated the immune system and increased the expression levels of apoptosis-related genes in HT29 tumor-bearing nude mice	Syed Najmuddin et al. (2020)
Prostate cancer	NDV/FMW	—	In nude mice bearing prostate tumors, the tumors injected with the supernatants of NDV/FMW-infected cells grew smaller than mock-treated tumors	Wang et al. (2020)
Glioblastoma	LaSota	Temozolomide	The combination of NDV-LaSota and temozolomide (TMZ) was effective in inducing apoptosis of glioma cells *in vitro* and *in vivo*	Bai et al. (2018)
	MTH-68/H	Mesenchymal stem cells	NDV induces dose-dependent cell death in glioma cells and a low level of apoptosis and inhibition of self-renewal in glioma stem cells	Kazimirsky et al. (2016)
Melanoma	NDV-NS1	Vanadyl sulfate	NDV, in combination with vanadyl sulfate, significantly increased the number of immune cells and resulted in rapid tumor regression in the B16-F10 mouse model	McAusland et al. (2021)
Clear cell renal cell carcinoma	AF2240	—	AF2240 induced the activation of the p38 MAPK/NF-κB/IκBα pathway in clear cell renal cell carcinoma, which resulted in cell death due to apoptosis	Ch’ng et al. (2015)
Orthotopic glioma	NDV HB1	—	NDV HB1 treatment significantly prolonged median survival (50%) and induced a long-term, tumor-specific immunological memory response	Koks et al. (2015)

^a^NDV(F3aa): the mutant NDV strain with the F cleavage site is modified with three amino acids; rL-hIFN-λ1: the recombinant NDV strain LaSota containing human IFN-λ1 gene; NDV-D90: the NDV strain that was isolated from natural sources in China; rNDV-18HL: the recombinant NDV Italien expressing the chimeric HAb18 antibody; rNDV/Anh-IL-2: the recombinant NDV *Anhinga* strain expressing IL-2 cytokine; NDV/FMW: the oncolytic NDV strain FMW; NDV AMHA1: the attenuated strain AMHA1 of NDV; AF2240: the NDV strain AF2240 that was isolated by the Malaysian Veterinary Research Institute in 1960; NDV HB1: the avirulent, non-lytic Hitchner B1 strain of NDV; R2B Mukteshwar: the R2B Mukteshwar strain of NDV; rAF-IL12: the recombinant NDV-AF2240 strain expressing IL-12 cytokine; MTH-68/H: the live attenuated oncolytic viral strain of the NDV; NDV-NS1: the recombinant fusogenic NDV expressing the influenza virus NS1 protein.

### Gastric Cancer

Gastric cancer is one of the most common malignancies in the digestive system and the second leading cause of cancer-related deaths worldwide, with an approximately overall 5-year survival rate of 30% (Ma et al., 2019; Zhao et al., 2019). Although significant development has been achieved in the treatment of gastric cancer, the prognosis of most patients with gastric cancer remains poor (Liu et al., 2020). Fortunately, the established NDV strains can effectively target and kill gastric cancer cells and activate immune responses (Ma et al., 2019; Liu et al., 2020; Wang et al., 2020), improving tumor treatment efficacy. To detect the oncolytic effect of NDV in gastric cancer, NDV–GFP was constructed by Wong et al. (2010)by inserting the enhanced green fluorescent protein (EGFP) gene, which is a reporter gene. Their study revealed that the GFP-expressing cells counterstained positive for the carcinoembryonic antigen expression in peritoneal lavage samples from gastric adenocarcinoma patients undergoing staging laparoscopy.

Furthermore, NDV–GFP may provide a more sensitive method than conventional cytology for detecting gastric cancer. Bu et al., 2019a and Bu et al., 2019breported that the recombinant LaSota strain expressing rL-RVG (rabies virus glycoprotein) suppressed nAChRs (nicotinic acetylcholine receptors) to reduce cell migration and EMT (epithelial to mesenchymal transition) in gastric cells. Moreover, rL-RVG suppressed the growth of gastric cancer subcutaneous tumor cells *in vivo* (Bu et al., 2019a).

As previously stated, NDV infection results in the release of multiple cytokines, including type I interferon (IFN), interleukin 1 (IL-1), and tumor necrosis factor-alpha (TNF-α) *in vitro* and *in vivo* (Shobana et al., 2013). Meanwhile, it has been confirmed that NDV strains armed with IFN or IL gene result in higher oncolytic efficacy in tumor cells (Mohamed Amin et al., 2019). For gastric cancer, the presence of various polymorphisms for genes coding IL-2, which is associated with poor prognosis in gastric cancer patients, might provide a therapeutic target to inhibit gastric cancer progression (Bai et al., 2014; Andersen et al., 2017). Thus, NDV has an excellent application prospect in immunotherapy for gastric cancer. In addition, it is reported that immune cells with improved survival and prognosis induce immunological memory in the gastric cancer cells and enhance tumor regression (Wong et al., 2010; Lam et al., 2011). In humans, the progression of gastric cancer is associated with the immune function of specific lymphocytes, such as NK cells (Liu et al., 2015; Subhash et al., 2015; Zhao et al., 2018). Cytokines are secreted after the NDV infection of tumor cells, which causes NK cells to become activated. The activated NK cells promote the cytokine release and the activation of other immune cell functions (Bie et al., 2016). To summarize, NDV has a severe toxic effect on gastric cancer cells. NDV-activated cytokines and immune cells, on the other hand, increase antitumor cytotoxic activity against gastric cancer cells and are subsequently predicted to cure gastric cancer.

### Liver Cancer

Liver cancer is the fifth most frequent cancer worldwide and the fourth contributor to cancer-related death globally (Mokrane et al., 2020; Ioannou, 2021). Curative resection or liver transplantation is the primary treatment for individuals with liver cancer, but therapeutic success is still poor (Huge et al., 2020). Therefore, novel treatment strategies are urgently needed to eliminate cancer cells effectively. Chen et al. (2016)demonstrated that a recombinant DNA vaccine containing the NDV HN gene inhibits hepatocellular carcinoma cell proliferation (HCC). Furthermore, it induces autophagy *via* the mitochondrial pathway *in vitro* and *in vivo*. This indicates that NDV-based cancer therapy is a promising candidate for liver cancer treatment.

In addition, modified NDV can significantly improve the therapeutic efficacy of NDV in the liver cancer model (Song et al., 2007; An et al., 2016). For some examples, NDV/Anh-TRAIL promotes the mouse liver cancer model to produce immune memory and protect mice from further malignant tumor challenges (Wu et al., 2017). IFN-stimulated gene (ISG)-12a mediates this process, but high basal ISG-12a may inhibit the replication and infection of NDV (Liu et al., 2014). An NDV vector with an L289A mutation inside the NDV F gene can improve NDV’s oncolytic action on HCC cells *in vitro* and *in vivo*, indicating promising potential (Altomonte et al., 2010). NDV expressing the chimeric antibody (cHAb18) against tumor-associated antigen CD147 inhibits HCC cell migration and invasion, induces tumor necrosis, and prolongs the survival time of mice (Wei et al., 2015).

Combination therapy, which offers more significant advantages than single-drug therapy, is becoming an increasingly essential aspect of anticancer therapies (Nastiuk and Krolewski, 2016; Martin and Bell, 2018). The combination of NDV therapy and traditional/non-traditional therapies may become a novel choice for HCC treatment. A recent study confirms that fludarabine as an adjuvant enhances the antitumor immunity of NDV-mediated HCC treatment (Meng et al., 2019). Also, the combination treatment of NDV with 5-FU has greater antitumor efficacy than treatment with NDV or 5-FU alone (Assayaghi et al., 2019). Although OVs can strongly trigger immune activation, a negative feedback is usually upregulated in TME (Reale et al., 2019). Meng et al. (2020)indicated that dichloroacetate improved NDV-mediated viral immunotherapy for HCC by reducing the negative immunological feedback and boosting viral replication. These data suggest that more research into the clinical transformation of NDV in immunotherapy for liver cancer is essential.

### Lung Cancer

Non–small cell lung cancers (NSCLCs) account for 85% of lung cancer cases and are the leading cause of cancer death (Tan et al., 2021). Increasing evidence suggests that NDV, in addition to direct oncolysis, mediates lung cancer cell proliferation by controlling the cell immune response (Ye et al., 2018; Shao et al., 2019). NDV-D90, an NDV strain isolated from natural sources, exerts an antiproliferative effect in A549 cells (human lung cancer cell lines) (Fu et al., 2011). Another NDV strain, RL-RVG, decreased tumor growth, subcutaneous tumor necrosis, tumor apoptosis, and increased clusters of differentiation (CD)3-/CD49 + NK cells in the tumor-bearing mice group (Yan et al., 2014). These findings emphasize the significance of NDV eliciting an antitumor immune response in lung cancer treatment. Furthermore, multiple studies have found that the occurrence and progression of cancer are linked to the deregulation of a range of microRNAs (miRNAs) (Hao et al., 2011; Che et al., 2020). The overexpression or suppression of miR-204 was substantially associated with NDV-induced oncolysis in A549 cells (Liang et al., 2021). Therefore, targeting some key miRNAs may provide a new direction for cancer therapy.

Autophagy is a defensive reaction to the cellular stress, such as viral infection. NDV inhibits mitophagy to increase viral replication by inhibiting intrinsic apoptosis (Meng et al., 2014). The induction of ICD determinants by NDV was significantly reduced when autophagy-related genes were knocked out in lung cancer cells (Ye et al., 2018). Moreover, the treatment of lung cancer spheroids with the autophagy inhibitor chloroquine increases NDV/FMW-induced cytotoxicity (Hu et al., 2015), indicating NDV may be a potential strategy for targeting lung cancer stem cells. These findings imply that NDV combined with autophagy modulators helps improve NDV’s cancer therapeutic activity.

### Breast Cancer

According to the most recent cancer statistics, breast cancer is currently the most frequent malignancy in women and one of the significant causes of death worldwide (Sung et al., 2021). Breast cancer can be classified based on immunohistochemical markers such as estrogen receptor (ER), progesterone receptor (PR), and human epidermal growth factor receptor 2 (HER2) (Xupeng et al., 2021). Despite considerable advancements in breast cancer treatment, patients with triple-negative breast cancer (TNBC) have restricted treatment options due to a lack of recognizable specific markers (Kalscheuer et al., 2019). NDV represents a great potential candidate in the treatment of breast cancer. According to the Kalantari et al. (2020)study, NDV killed breast cancer cells by triggering the intrinsic apoptotic pathway, characterized by elevated Bax, caspase-9, and caspase-3. NDV-D90 induced apoptosis by differentially modulating the expression of ERα and GPER in ER-positive/negative breast cancer cells exposed to estrogen, respectively (Shan et al., 2021).

Furthermore, NDV-AF2240, as an ideal inducer of apoptosis, induces the apoptosis of breast cancer cells and is more cytotoxic to breast cancer than other NDV strains (Raihan et al., 2019). These results suggest that NDV promotes breast tumor regression *via* apoptotic-dependent pathways. In addition, the breast cancer cells infected with NDV showed a significant decrease in glycolysis activity (Al-Ziaydi et al., 2020a). NDV also plays an essential role in the combined treatment of breast cancer. The 2-DG (2-deoxyglucose), a kind of glucose analog in combination with NDV, showed more significant tumor growth inhibition than in a single treatment (Al-Shammari et al., 2019). D-Mannoheptulose, a particular hexokinase inhibitor, was employed by Ahmed et al. to prevent glycolysis and increase the antitumor activity of NDV (Al-Ziaydi et al., 2020b). The hemagglutinin–neuraminidase (HN) protein of NDV enables NDV to target breast cancer cells (Al-Ziaydi et al., 2020b) effectively. Therefore, NDV has a promising future in the treatment of breast cancer.

### Other Cancers

As an oncolytic agent, NDV has been reported in other types of cancers (Table 1), including cervical cancer (Keshavarz et al., 2020a), prostate cancer (Wang et al., 2020), colorectal cancer (Song et al., 2019), and glioblastoma (Abdullah et al., 2014). Cancer is a dynamic disease (Dagogo-Jack and Shaw, 2018), so there are significant differences in cancer cells from different tissue sources, even if there is phenotypic and functional heterogeneity among cancer cells in the same tumor (Meacham and Morrison, 2013). In addition, NDV has other killing mechanisms in different cell lines (Ginting et al., 2019; Li et al., 2019), suggesting that we should carry out the targeted treatment when developing NDV therapy. MSCs (mesenchymal stem cells) represent a potential delivery method (Uder et al., 2018). For instance, Mohsen K et al. found that an MSC-engineered system significantly reduced tumor growth, enhancing CD8^+^ T-cell cytolysis responses and splenic cytokine responses. This finding demonstrates that MSCs expressing oncolytic NDV may be a viable method for cancer immunotherapy (Keshavarz et al., 2020b).

## Clinical Application of Newcastle Disease Virus

Increasing clinical evidence indicates that oncolytic NDV as a therapeutic agent (a type of immunotherapy) can eliminate glioma, metastatic cancer, and advanced solid tumor cells while stimulating patients’ immune systems as well (Table 2).

**TABLE 2:** NDV strains for different cancer treatments in clinical trials.

NDV strain	Reference	Cancer	Phase	Patient	Outcome
ATV-NDV-αHN-αCD28	Schirrmacher et al. (2015)	Colorectal cancer	Phase I	Fourteen patients whom all suffered from stage IV colorectal cancer (with distant metastases)	The decrease in CEA in four patients and the partial response of metastases in four patients were observed. Seven patients were still alive in 2009
ATV-NDV	Steiner et al. (2004)	Glioblastoma	Phase III	Twenty-three patients with a pathologically confirmed glioblastoma	91% of vaccinated patients survived 1 year, 39% survived 2 years, and 4% were long-term survivors
ATV-NDV	Karcher et al. (2004)	Head and neck squamous cell carcinoma	Phase III	Twenty patients with pathologically confirmed head and neck squamous cell carcinoma	Percentages of survival of vaccinated patients with stage III and stage IV tumors (*n* = 18) were 61% at 5 years
MTH-68/H	Csatary et al. (2004)	Glioblastoma multiforme	Phase I	Four patients with advanced high-grade glioma	All patients (*n* = 4) with advanced high-grade glioma were treated with MTH-68/H, resulting in survival rates of 5–9 years
NDV-73T	Batliwalla et al. (1998)	Melanoma	Phase II	Fifty-one patients with AJCC stage III melanoma	The 10-year survival of the NDV-73T group of patients was more than 60%, and the overall 15-year survival was 55%, with no adverse reactions
NDV-HUJ	Freeman et al. (2006)	Glioblastoma multiforme	Phase I/II	Eleven patients with glioblastoma multiform based on histology	Toxicity was minimal, with grade I/II constitutional fever seen in five patients. One patient achieved a complete response (1/11)

^a^ATV-NDV: the NDV-modified autologous tumor vaccine; ATV-NDV-αHN-αCD28: the ATV-NDV strain expressing the anti-CD28 fusion protein, coupled to viral HN anchor molecules; NDV-73T: the mesogenic strain of NDV.; MTH-68/H: the live attenuated oncolytic viral strain of the NDV; NDV-HUJ: he NDV strain isolated from naturally attenuated B1 NDV vaccine strain.

The major NDV strains evaluated for direct human injection are PV-701 (Pecora et al., 2002), 73-T (Cassel and Garrett, 1965), MTH-68/H (Csatary et al., 1999), and ATV-NDV (Schirrmacher and Fournier, 2009), which are lytic and HUJ (Yaacov et al., 2008), which is non-lytic. In 1964, Wheelock and Dingle (1964) first reported the use of NDV in the treatment of human cancer. After a patient with acute myeloid leukemia was continuously inoculated with the NDV Hickman strain, the number of leukemia cells decreased rapidly, and the symptoms improved, which lasted for nearly 2 weeks (Wheelock and Dingle, 1964). In the following year, a study by William Cassel and his colleagues showed that patients with stage II and III melanoma resected with NDV-73T strain oncolysis were vaccinated with improved overall survival (Cassel et al., 1977; Murray et al., 1977; Cassel et al., 1983). Long-term follow-up of these patients showed a 10-year survival rate of more than 60% and a 15-year survival rate of 55% compared with historical controls (Cassel and Murray, 1992; Batliwalla et al., 1998). This is the early use of NDV-based tumor vaccines for active tumor–specific immunity. Liang et al. later confirmed using an autologous NDV–modified tumor cell vaccination to treat gastrointestinal cancers. They compared 310 patients with stage I–IV colorectal cancer who received resection and immunotherapy with 257 patients who received chemotherapy with resection alone. The median overall survival of the vaccine group was more than 7 years, while that of the resection group was 4.46 years (Liang et al., 2003; Burman et al., 2020). In non-controlled experiments, adjuvant immunization with autologous NDV–modified cancer cells was safe and advantageous.

Immune checkpoint inhibitors are one of the most promising agents in tumor therapy in recent years (Nettelbeck et al., 2021). Durvalumab is a selective, high-affinity, human IgG1 monoclonal antibody that blocks programmed death-ligand 1 (PD-L1) binding to programmed death 1 (PD-1) (Stewart et al., 2015). Recombinant NDV (MEDI5395) expressing granulocyte-macrophage colony-stimulating factor (GMCSF), based on the strain NDV-73T, is being evaluated with intravenous administration (NCT03889275) in conjunction with durvalumab in patients with various advanced malignant tumors (Burke et al., 2020). Other recombinant NDVs are at different stages of development and are expected to enter clinical practice in the next few years. Meanwhile, NDV can be armed with foreign genes *via* the reverse genetic technology to achieve more effective and diverse antitumor effects. The combination of genetic engineering NDV with computational approaches may be beneficial to enhance the efficacy of clinical cancer treatment (Lathwal et al., 2020).

## Conclusion

The tumor is a recalcitrant disease that poses a severe threat to human life and health. NDV acts as a potent oncolytic agent by causing apoptosis, autophagy and necrosis in tumor cells, limiting cell metabolism, and generating a series of immunological responses. At the same time, it has essentially no effect on human normal cells. NDV is also one of the few viruses that have been found to produce partial or even complete responses when treated with a single medication. The persistence of these responses suggests that the virus’s therapeutic effect may depend not only on direct oncolysis but also on the virus’s potential to promote long-term immunity. With the development of virotherapy, the activation of the immune responses through cancer virotherapy may eradicate tumors. NDV currently shows great promise in preclinical and clinical trials.

NDV replication occurs in the cytoplasm and does not integrate into the genome of the host, maintaining the safety of the parental virus. The oncolytic property of NDV is either lytic or non-lytic that only infect cells with a disturbed interferon system, which improves the safety of NDV as a vaccine. NDV does not need to be armed with foreign genes to have a strong antitumor effect and stable expression of foreign genes. The combination of NDV virus therapy and traditional/new tumor treatment techniques has been reported and has broad application prospects. However, many questions about NDV therapy, such as those about other OVs, remain unresolved, including the practical techniques of administration, the best genetic engineering strategies, the therapeutic sequence of immune checkpoint inhibitors, and the best combination partners. There is currently no conventional optimum method for how and when patients should use the virus. The tumor microenvironmental barrier and the cytoplasmic matrix of solid tumors may interfere with and inhibit virus invasion and replication, reducing its oncolytic action. Excessive foreign genes will affect the replication of NDV. Moreover, the preparation of NDV needs deep purification to obtain clinical-grade virus preparation.

Cancer patients are usually immunocompromised, while immunocompromised patients may benefit more from OV therapy. For example, cancer patients infected with COVID-19 have low levels of antibodies against the spike protein. An oncolytic vaccine based on the spike protein not only has a strong antitumor effect but also may be beneficial to the prevention of COVID-19. Further understanding of the immunological system may help develop more effective oncolytic NDV and the elimination of the NDV treatment barrier in solid tumors. The combination of NDV therapy and traditional/non-traditional therapies may become a novel choice for cancer treatment. Combining NDV viral therapy with existing immunotherapy, which uses NDV’s effect on the immune response, may result in a higher antitumor effect. As a result, NDV is likely to be an ideal tumor therapeutic agent in the future.
